# Diagnostic performance of metagenomic sequencing in patients with suspected infection: a large-scale retrospective study

**DOI:** 10.3389/fcimb.2024.1463081

**Published:** 2024-09-06

**Authors:** Ziyang Li, Li Tan, Jialiang Zhang, Qichen Long, Zhiyang Chen, Zhongyuan Xiang, Weimin Wu, Zhe Guo, Huifang Liu, Bingxue Hu, Bin Yang, Min Hu

**Affiliations:** ^1^ Center for Clinical Molecular Diagnostics, The Second Xiangya Hospital, Central South University, Changsha, Hunan, China; ^2^ Department of Laboratory Medicine, The Second Xiangya Hospital, Central South University, Changsha, Hunan, China; ^3^ Center for Infectious Diseases, Vision Medicals Co., Ltd, Guangzhou, Guangdong, China

**Keywords:** metagenomic next generation sequencing, infectious diseases, sensitivity, clinical diagnosis, pathogens

## Abstract

**Background:**

Metagenomic next-generation sequencing (mNGS) has been widely reported to identify pathogens in infectious diseases (IDs). In this work, we intended to investigate the diagnostic value and clinical acceptance of paired-samples mNGS as compared to the culture method.

**Methods:**

A total of 361 patients with suspected infection were retrospectively included. With reference to the clinical diagnosis, we compared the diagnostic performance and clinical acceptance in pathogen detection between mNGS and culture tests. Moreover, the pathogen concordance of paired blood and respiratory tract (RT) samples in mNGS assay was investigated.

**Results:**

Among 511 samples, 62.04% were shown to be pathogen positive by mNGS, and that for clinical diagnosis was 51.86% (265/511). When compared to culture assay (*n* = 428), mNGS had a significantly higher positivity rate (51.87% vs. 33.18%). With reference to the clinical diagnosis, the sensitivity of mNGS outperformed that of culture (89.08% vs. 56.72%). Importantly, mNGS exhibited a clinically accepted rate significantly superior to that of culture. In addition, the mNGS result from 53 paired blood and RT samples showed that most pairs were pathogen positive by both blood and RT, with pathogens largely being partially matched.

**Conclusion:**

Through this large-scale study, we further illustrated that mNGS had a clinically accepted rate and sensitivity superior to those of the traditional culture method in diagnosing infections. Moreover, blood and paired RT samples mostly shared partial-matched positive pathogens, especially for pathogens with abundant read numbers in RT, indicating that both blood and RT mNGS can aid the identification of pathogens for respiratory system infection.

## 1 Introduction

With over 8 million deaths worldwide, infectious diseases (IDs) remain the leading cause of morbidity and mortality in terms of high frequency of occurrence (Standing up to infectious disease, 2019). Moreover, a resurgence of public health insurance failures for diseases we have managed has also been caused by the appearance of novel IDs, accelerated transmission of established pathogens, and increased drug resistance over the past few decades, rendering our present antimicrobials ineffective (McArthur, 2019; Makam and Matsa, 2021). Thus, fighting IDs remains a priority in the 21st century, necessitating multifaceted strategies that make use of innovative tools for infection tracking, prevention, diagnosis, and, ultimately, treatment.

However, the fact that a wide variety of organisms can cause illnesses that are clinically similar makes accurate diagnosis difficult. A battery of tests is frequently applied in order to attempt to establish a diagnosis using current methods including culturing, serological assays, and nucleic acid amplification techniques (Miller et al., 2018). Importantly, despite the advances of cutting-edge technologies used to diagnose IDs, such as polymerase chain reaction (PCR) panels and 16S ribosomal DNA Sanger sequencing, the etiology of IDs remains unknown in up to 60% of cases, depending on the clinical condition (Schlaberg et al., 2017; Nanotechnology for infectious diseases, 2021).

Unbiased metagenomic next-generation sequencing (mNGS) has emerged as a promising single, universal pathogen detection method for ID diagnostics (Simner et al., 2018). Previous studies have demonstrated that mNGS outperformed culture in diagnosing IDs, manifested as a broader pathogen spectrum and superior sensitivity and specificity (Miao et al., 2018; Duan et al., 2021; Tao et al., 2022; Xiao et al., 2023), indicating its advantages in diagnosing suspected infections. However, whether the results of mNGS were accepted by clinics and the feasibility of blood mNGS in aiding pathogen identification in respiratory system infection remain elusive. In the current work, using a large-scale sample number and a broad array of sample types, we intended to assess the performance and clinical acceptance of paired-samples mNGS in pathogen detection and ID diagnosis in clinical practice.

## 2 Materials and methods

### 2.1 Study patients

**Figure 1:**
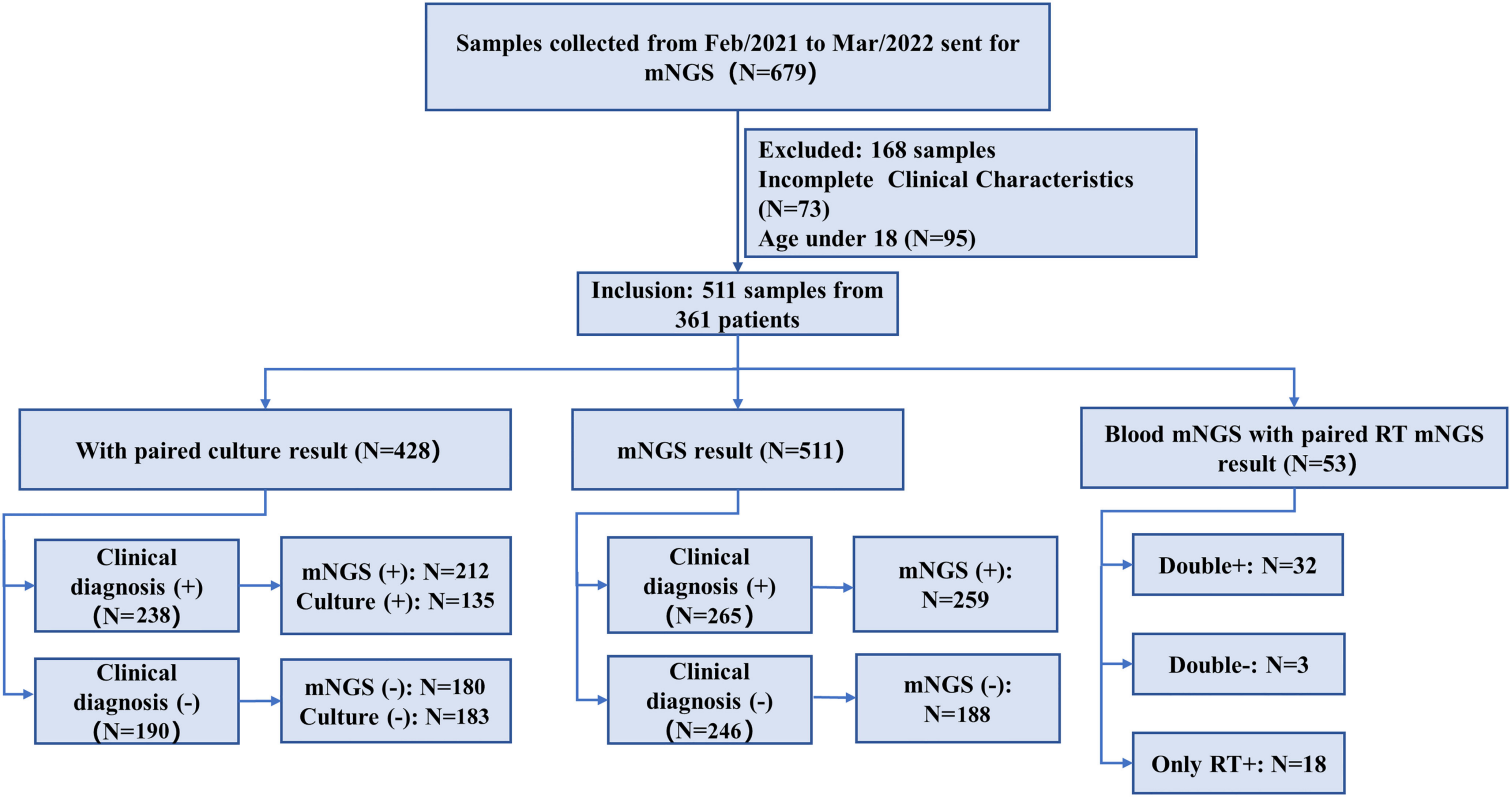
Flowchart of sample selection and classification. From 679 samples, a total of 511 were selected for further analysis. Samples were divided into clinical diagnosis positive and clinical diagnosis negative based on the retrospective composite diagnosis.

We retrospectively reviewed 361 patients with suspected infection at the Second Xiangya Hospital, Central South University, China, between February 2021 and May 2022. A total of 511 samples were included in the current study, according to our inclusion/exclusion criteria (**Figure 1**). Specimens were subjected (or not) to an mNGS assay (Illumina Nextseq 500Dx) as well as a culture test according to the physician’s order sheet after hospitalization in the same day. An expert group consisting of a microbiologist, a molecular biologist, and ID physicians retrospectively adjudicated patients’ final clinical diagnoses according to the composite diagnostic criteria and patients’ characteristics. The expert group members independently assessed each sample, and an accordant conclusion was reached through discussion when their judgments were inconsistent. Based on the final clinical diagnoses of each sample, samples were categorized into clinically diagnosis positive and clinically diagnosis negative, and the corresponding patients were diagnosed with or without definite IDs.

This study was approved by the Ethics Committee of the Second Xiangya Hospital, Central South University (LYF2022229). The study was considered exempt from informed consent as it was a retrospective observational cohort study.

### 2.2 mNGS sequencing and analysis

DNA was extracted from all samples using a QIAamp^®^ UCP Pathogen DNA Kit (Qiagen) according to the manufacturer’s instructions. Human DNA was removed using Benzonase (Qiagen) and Tween20 (Sigma). The QIAamp UCP pathogen mini kit (Qiagen, Valencia, CA, USA) was applied to extract total RNA. According to the manufacturer’s recommendations, a total of 1 µL of sample was processed with Turbo DNase (Life Technologies, USA) to deplete the host DNA background. RNA was reversely transcribed and amplified by the Ovation RNA-Seq system (NuGEN, CA, USA). Following fragmentation, the library was constructed using Ovation Ultralow System V2 (NuGEN, CA, USA) and was sequenced on Illumina Nextseq 550 (single-end 75 bp) (Ren et al., 2018). For negative controls, peripheral blood mononuclear cell samples with 10^5^ cells/mL from healthy donors in parallel were prepared with each batch, using the same protocol, and sterile deionized water was extracted alongside the specimens to serve as non-template controls (NTCs) (Miller et al., 2019). Raw sequencing data were processed using fastp (Chen et al., 2018) to remove reads containing adapters or ambiguous “N” nucleotides and low-quality reads. Low-complexity reads were removed by Kcomplexity with default parameters (Bolger et al., 2014). Human sequence data were identified and excluded by mapping to a human reference genome (hg38) using Burrows–Wheeler Aligner software (Li and Durbin, 2009). Microbial reads were then aligned to the database with SNAP v1.0 beta.18. Approximately 20 million reads were generated for each sample. For pathogen with background reads in negative control, a positive detection was reported for a given species or genus if the reads per million (RPM) ratio was ≥10, where the RPM ratio was defined as the RPM_sample_/RPM_NTC_. For pathogen without background reads in negative control, RPM was set as ≥0.05. A penalty of 5% and 10% was used for species and genus, respectively (Miller et al., 2019).

### 2.3 Definition of sensitivity, specificity, positive predictive value, negative predictive value, and clinically accepted rate

With reference to clinical diagnosis, sensitivity was defined as [(positive detection in clinical diagnosis positive)/(samples in clinical diagnosis positive)], while specificity was defined as [(negative detection in clinical diagnosis negative)/(samples in clinical diagnosis negative)]. Positive predictive value (PPV) was defined as [(positive detection in clinical diagnosis positive)/(total positive detection)]. In contrast, negative predictive value (NPV) was defined as [(negative detection in clinical diagnosis negative)/(total negative detection)]. Based on the clinical diagnosis of each sample, whether the result of mNGS or culture was accepted by the clinic was judged according to **Supplementary Table 1**, and the clinically accepted rate was defined as [(clinically accepted samples)/(clinically accepted samples+ clinically unaccepted samples)×100%].

### 2.4 Statistical analysis

Continuous variables were presented as medians and quartile (first quartile, third quartile), and categorical variables were expressed as counts and percentages. The categorical variables were compared using the chi-square test. *p* < 0.05 was considered significant. Wilcoxon paired rank sum test was applied for comparing the read numbers between paired samples. Statistical analyses were performed using SPSS version 23.0 (SPSS, Inc., Chicago, IL, USA).

## 3 Results

### 3.1 Samples and patient characteristics

Among 361 patients, a total of 241 patients (66.76%) were male and the median age was 57 years old. Hypertension, chronic cardiac disease, and chronic kidney disease were found in 29.36%, 21.33%, and 20.22% of patients, respectively (**Table 1**). The majority of patients (245, 67.87%) provided one sample, 98 patients provided two samples, and the remaining patients provided three to nine samples. In total, 511 samples were included in this study, and most were bronchoalveolar lavage fluid (BALF, *n* = 194, 37.96%), followed by blood (180, 35.23%). Meanwhile, 51 (9.98%), 19 (3.72%), and 19 (3.72%) samples were collected from cerebrospinal fluid (CSF), tissue, and sputum, respectively (**Table 1**). Other samples (*n* = 48, 9.39%) included pleural and ascites, joint fluid, and drainage fluid, which were categorized into the “other” group in the following analysis. According to the clinical diagnosis of each sample they tested, there were 211 of 361 patients (58.45%) diagnosed with definite IDs, and the predominant ID was respiratory system infection, followed by multifocal infection and bloodstream infection (**Table 1**).

**Table 1:** Demographic characteristics of patients and samples.

Clinical features	Numbers
Case number	361
**Sex, male, *n* (%)**	241 (66.76)
**Age, years median (Q1, Q2)**	57 (47, 68)
Medical history, *n* (%)	
Chronic respiratory disease	45 (12.47)
Hypertension	106 (29.36)
Diabetes mellitus	59 (16.34)
Chronic kidney disease	73 (20.22)
Tumor	60 (16.62)
Chronic cardiac disease	77 (21.33)
Chronic hepatic disease	64 (17.73)
Cerebrovascular disease	46 (12.74)
Sample number per case, *n* (%)	*n* = 361
1	245 (67.87)
2	98 (27.15)
3	11 (3.05)
4	3 (0.83)
5	2 (0.55)
6	1 (0.28)
9	1 (0.28)
Sample type, *n* (%)	*n* = 511
BALF	194 (37.96)
Blood	180 (35.23)
CSF	51 (9.98)
Sputum	19 (3.72)
Tissue	19 (3.72)
Pleural and ascites	15 (2.94)
Joint fluid	11 (2.15)
Urine	6 (1.17)
Drainage fluid	3 (0.59)
Secretions	3 (0.59)
Vitreous humor	2 (0.39)
Aqueous humor	2 (0.39)
Swab	2 (0.39)
Bone marrow	1 (0.2)
Bile	1 (0.2)
Pus	1 (0.2)
Pericardial effusion	1 (0.2)
Infectious types, *n* (%)	*n* = 210
Respiratory system infection	112 (53.33)
Multifocal infection	38 (18.10)
Bloodstream infection	22 (10.48)
Skin and soft tissue infection	13 (6.19)
Central nervous system infection	6 (2.86)
Intra-abdominal Infection	6 (2.86)
Urinary system infection	6 (2.86)
Bone and joint infection	3 (1.43)
Eye infection	3 (1.43)
Cardiovascular system infection	1 (0.48)

BALF, bronchoalveolar lavage fluid; CSF, cerebrospinal fluid.

### 3.2 Comparison of positive rate for mNGS results and clinical diagnosis

**Figure 2:**
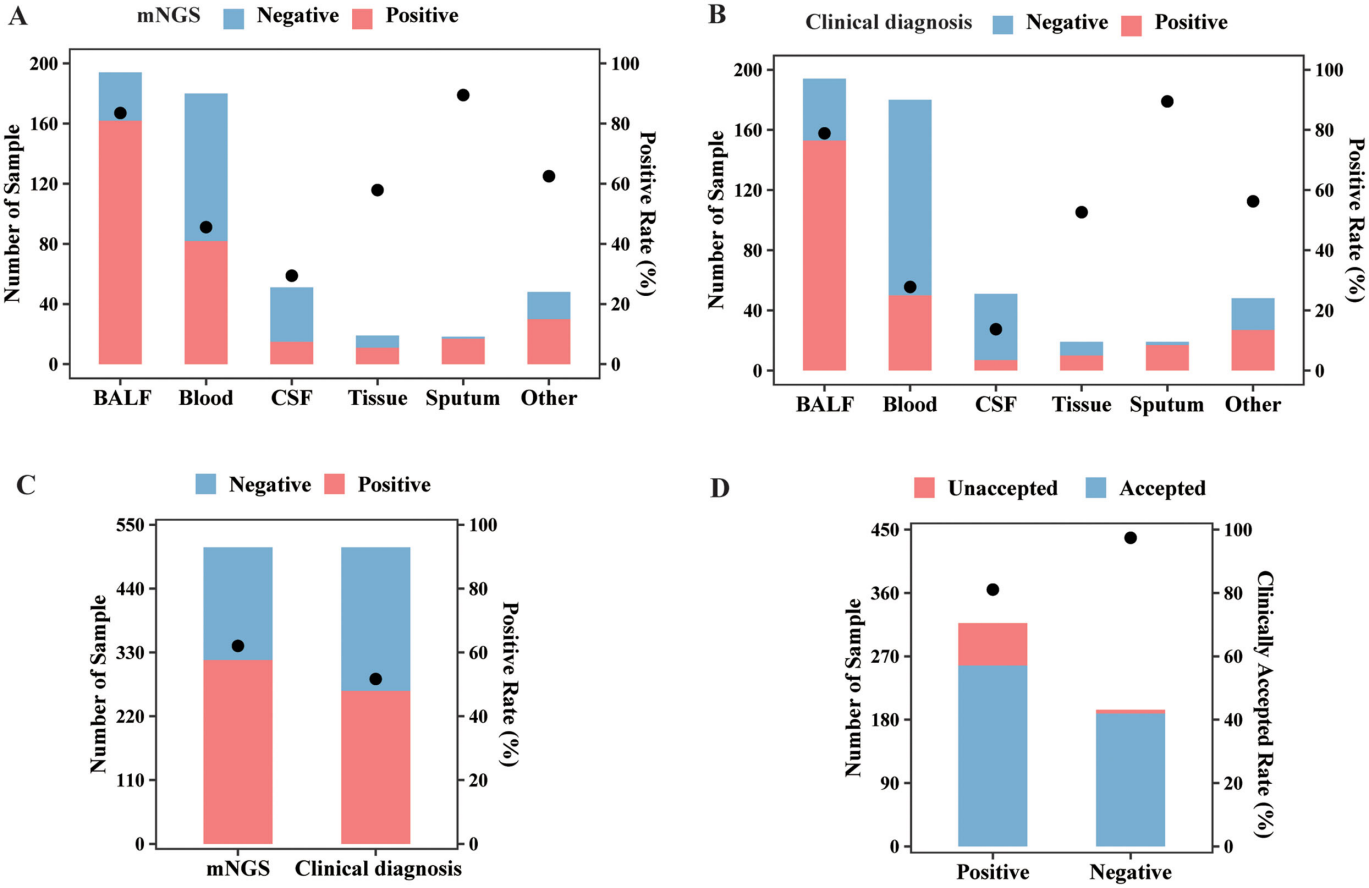
mNGS and clinical diagnosis results of different sample types. **(A)** The sample numbers and positive rate of the mNGS assay in different sample types. **(B)** The sample numbers and positive rate of clinical diagnosis in different sample types. **(C)** The sample numbers and overall positive rate of mNGS and clinical diagnosis. **(D)** The numbers of clinically accepted and unaccepted samples and accepted rate for mNGS-positive and -negative results. Abbreviation: mNGS, metagenomic next-generation sequencing.

Among all sample types, the highest positive rate of mNGS test was detected in sputum (89.47%), followed by BALF (83.51%), tissue (57.89%), blood (45.56%), and CSF (29.41%) by mNGS (**Figure 2A**). Based on the clinical diagnosis, the highest positive rate (**Figure 2B**) was also observed in sputum, which was 89.47%, followed by BALF (78.87%). The positive rate for clinical diagnosis was significantly lower than that for mNGS in BALF (78.87% vs. 83.51%, *p* < 0.05), blood (28.33% vs. 45.56%, *p* < 0.001), and CSF (13.73% vs. 29.41%, *p* < 0.01). As a result, the overall positive rate for mNGS was 62.04% (317/511), also significantly higher than that for clinical diagnosis (51.86%, 265/511, *p* < 0.001, **Figure 2C**). Furthermore, the clinically accepted rate for mNGS-positive and -negative results was 81.07% (257/317) and 97.42% (189/194), respectively, with significant difference (*p* < 0.001, **Figure 2D**). Therefore, mNGS obtained an overall accepted rate of 87.28% (446/511).

### 3.3 Comparison of diagnostic performance for mNGS and culture

**Figure 3:**
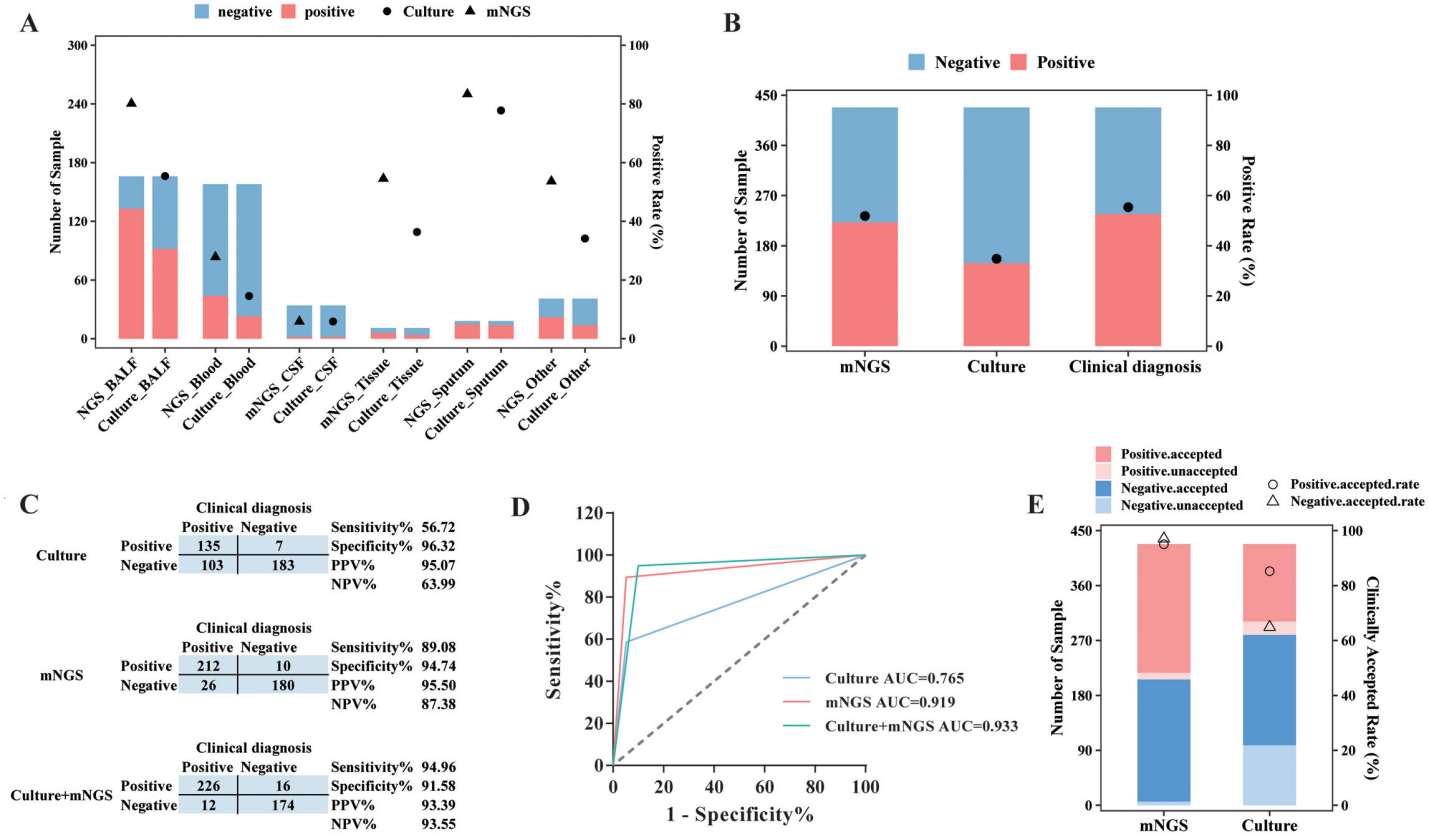
Diagnostic performance comparison between mNGS and culture. **(A)** The sample numbers and positive rate of mNGS and culture for different types of samples. **(B)** The sample numbers and overall positive rate of mNGS, culture, and clinical diagnosis for all samples. **(C)** Contingency tables formatted showing the respective diagnostic performance of mNGS and culture assays with reference to clinical diagnosis. **(D)** The receiver operator characteristic curve for mNGS and culture assays with reference to clinical diagnosis. **(E)** The numbers of clinically accepted and unaccepted samples and accepted rate for mNGS and culture-positive and -negative results. mNGS, metagenomic next-generation sequencing; NPV, negative predictive value; PPV, positive predictive value; AUC, area under the curve.

Of these 511 specimens, a total of 428 samples had matched culture results, and the positive rate and diagnostic performance of mNGS and culture were compared, after excluding detected viruses in mNGS results. mNGS showed a positive result in 80.12% (133/166) of BALF samples, 27.85% (44/158) of blood samples, 5.88% (2/34) of CSF samples, 54.55% (6/11) of tissue samples, and 83.33% (15/18) of sputum samples (**Figure 3A**). In addition, based on culture results, 52.41% of (87/166) BALF samples, 13.92% (22/158) of blood samples, 2.94% (1/34) of CSF samples, 36.36% (4/11) of tissue samples, and 77.78% (14/18) of sputum samples were confirmed to be pathogen positive (**Figure 3A**). The positive rate for BALF and blood from mNGS results was remarkably higher than that for culture (*p* < 0.01).

As for all 428 samples, the overall positive rate for mNGS was 51.87% (222/428), significantly higher than that for culture (33.18%, 142/428, *p* < 0.001, **Figure 3B**). Moreover, the clinical diagnosis regarded 55.61% (238/428) of samples as pathogen positive, with a larger positive rate than mNGS and culture (*p* < 0.05, **Figure 3B**). Taking clinical diagnosis as the gold standard, the sensitivity and specificity of culture assay were 56.72% and 96.32%, respectively, with an AUC of 0.765. As for the mNGS test, the sensitivity was 89.08%, significantly higher than that of culture (*p* < 0.001), with an AUC of 0.919. After combining mNGS and culture results, the sensitivity increased to 94.94%, and the AUC was 0.933 (**Figures 3C,D**). Additionally, although the diagnostic performance of mNGS and culture varied largely among different sample types, it could be observed that mNGS showed better sensitivity and AUC than culture, no matter what the sample type was (**Table 2**).

**Table 2:** Diagnostic performance of mNGS and culture tests in infections with reference to clinical diagnosis in different sample types.

Sample type	Tests	Sensitivity% (95% CI)	Specificity% (95% CI)	PPV% (95% CI)	NPV% (95% CI)	AUC (95% CI)
Total (*N* = 428)	Culture	56.72 (52.03–61.42)	96.32 (94.53–98.1)	95.07 (93.02–97.12)	63.99 (59.44–68.53)	0.765 (0.72–0.81)
	mNGS	89.08 (86.12–92.03)	94.74 (92.62–96.85)	95.5 (93.53–97.46)	87.38 (84.23–90.52)	0.919 (0.89–0.949)
	Culture+mNGS	94.96 (92.88–97.03)	91.58 (88.95–94.21)	93.39 (91.03–95.74)	93.55 (91.22–95.88)	0.933 (0.905–0.961)
BALF (*N* = 166)	Culture	63.97 (56.67–71.27)	100 (100–100)	100 (100–100)	37.97 (30.59–45.36)	0.82 (0.757–0.882)
	mNGS	95.59 (92.46–98.71)	90 (85.44–94.56)	97.74 (95.49–100)	81.82 (75.95–87.69)	0.928 (0.862–0.994)
	Culture+mNGS	99.26 (97.97–100.56)	90 (85.44–94.56)	97.83 (95.61–100.04)	96.43 (93.61–99.25)	0.946 (0.883–1)
Blood (*N* = 158)	Culture	33.33 (25.98–40.68)	95.33 (92.04–98.62)	77.27 (70.74–83.81)	75 (68.25–81.75)	0.643 (0.545–0.742)
	mNGS	74.51 (67.71–81.31)	94.39 (90.81–97.98)	86.36 (81.01–91.71)	88.6 (83.64–93.55)	0.845 (0.768–1)
	Culture+mNGS	88.24 (83.21–93.26)	90.65 (86.12–95.19)	81.82 (75.8–87.83)	94.17 (90.52–97.83)	0.894 (0.834–0.955)
CSF (*N* = 34)	Culture	14.29 (2.52–26.05)	100 (100–100)	100 (100–100)	81.82 (68.85–94.78)	0.571 (0.314–0.829)
	mNGS	28.57 (13.39–43.76)	100 (100–100)	100 (100–100)	84.38 (72.17–96.58)	0.643 (0.382–0.904)
	Culture+mNGS	28.57 (13.39–43.76)	100 (100–100)	100 (100–100)	84.38 (72.17–96.58)	0.643 (0.382–0.904)
Tissue (*N* = 11)	Culture	66.67 (38.81–94.52)	100 (100–100)	100 (100–100)	71.43 (44.73–98.13)	0.83 (57.57–100)
	mNGS	100 (100–100)	100 (100–100)	100 (100–100)	100 (100–100)	1 (1–1)
	Culture+mNGS	100 (100–100)	100 (100–100)	100 (100–100)	100 (100–100)	1 (1–1)
Sputum (*N* = 18)	Culture	81.25 (63.22–99.28)	50 (26.9–73.1)	92.86 (80.96–104.75)	25 (5–45)	0.656 (0.21–1)
	mNGS	93.75 (82.57–104.93)	100 (100–100)	100 (100–100)	66.67 (44.89–88.44)	0.969 (0.887–1)
	Culture+mNGS	100 (100–100)	50 (26.9–73.1)	94.12 (83.25–104.99)	100 (100–100)	0.75 (0.289–1)
Other (*N* = 41)	Culture	59.09 (44.04–74.14)	94.74 (87.9–101.57)	92.86 (84.97–100.74)	66.67 (52.24–81.1)	0.769 (0.621–0.917)
	mNGS	95.45 (89.08–101.83)	94.74 (87.9–101.57)	95.45 (89.08–101.83)	94.74 (87.9–101.57)	0.951 (0.873–1)
	Culture+mNGS	100 (100–100)	89.47 (80.08–98.87)	91.67 (83.21–100.13)	100 (100–100)	0.947 (0.865–1)

BALF, bronchoalveolar lavage fluid; CSF, cerebrospinal fluid; PPV, positive predictive value; NPV, negative predictive value; CI, confidence interval.

We next compared the clinically accepted rate between mNGS test and culture. As demonstrated in **Figure 3E**, 95.05% (211/222) of positive results and 96.60% (199/206) of negative results of mNGS were accepted by the clinic. In addition, the culture assay achieved an accepted rate of 93.66% (133/142) for its positive results and 65.73% (188/286) for its negative results. We found that the clinically accepted rate of the mNGS assay was significantly superior to culture, regardless of the positive and negative results (*p* < 0.01, **Figure 3E**).

### 3.4 mNGS exhibited advantages in pathogen detection compared to culture assay

**Figure 4:**
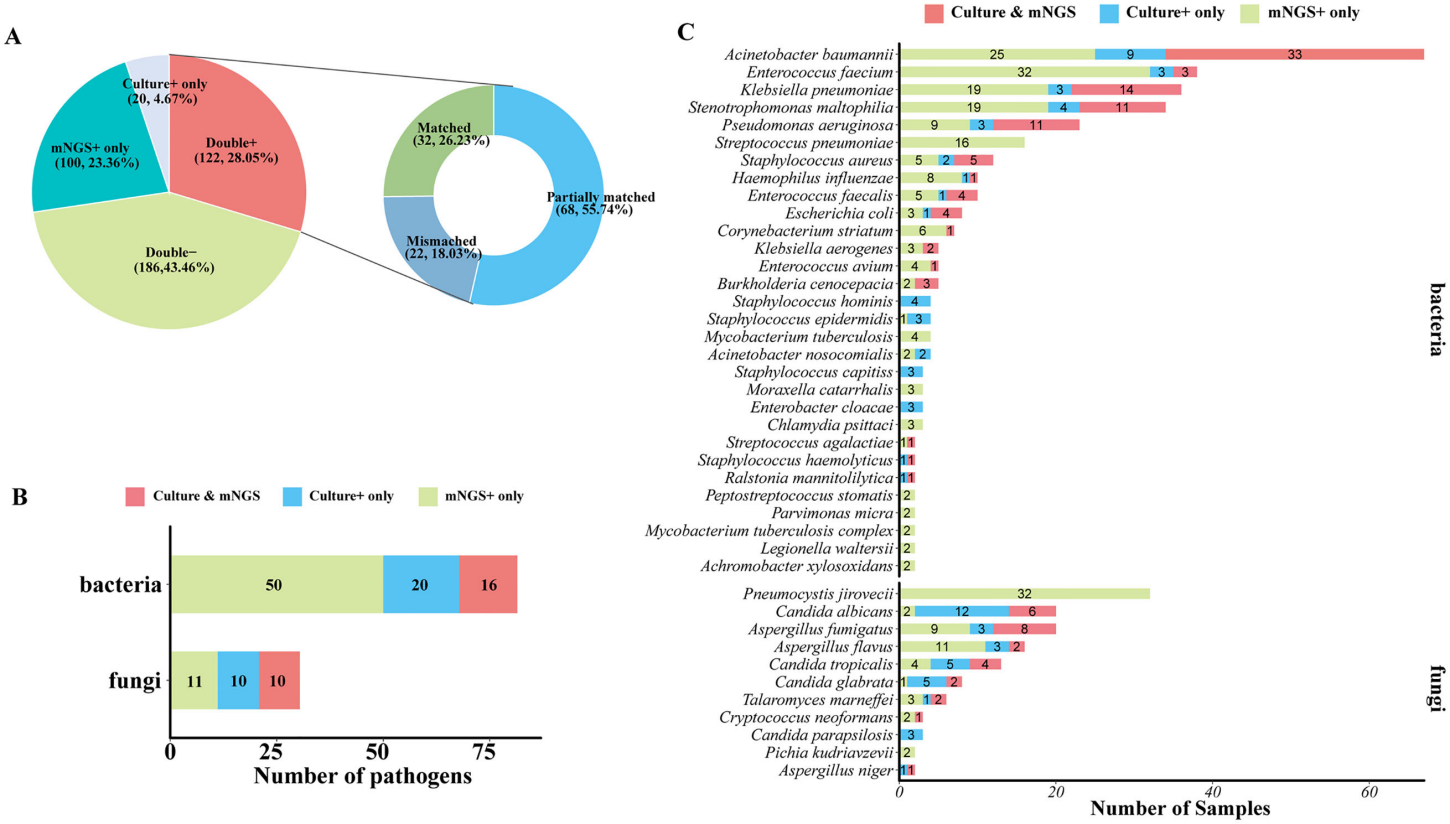
Comparison of mNGS and culture test for pathogen detection. **(A)** Pie chart demonstrating the positivity distribution of mNGS and culture for all samples. The double-positive samples were further categorized as matched (detected pathogens were identical), mismatched (no overlap of detected pathogens), and partially matched (at least one overlap of pathogens was observed). **(B)** The number of bacterial pathogens and fungal pathogens at the species level that were detected by culture only, mNGS only, or mNGS and culture. **(C)** The number of samples that were shown to be positive of corresponding bacterial pathogens and fungal pathogens at the species level by culture only, mNGS only, or mNGS and culture.

As for detected pathogen comparison between mNGS and culture (**Figure 4A**), they both showed a positive result in 122 of 428 samples (28.05%) and were both negative in 186 of 428 (43.46%) samples. A total of 100 (23.36%) samples were detected to be positive in the mNGS assay only, and 20 (4.67%) samples were positive in the culture test only. For 122 double-positive samples, the results of mNGS and culture completely matched (positive pathogens were identical) in 32 (26.23%) samples, partially matched (shared at least one positive pathogen) in 68 (55.74%) samples, but mismatched (positive pathogens were completely inconsistent) on pathogen identification in 22 (18.03%) samples (**Figure 3D**). In addition, among 100 mNGS-positive-only samples, 91 samples (91%) were confirmed to be positive by clinical diagnosis, including 48 of 51 (94.12%) BALF, 28 of 33 (84.85%) blood, 1 CSF (100%), 2 tissue (100%), 3 sputum (100%), and 9 of 10 (90%) other samples (**Supplementary Figure 1A**). A total of 137 pathogens were detected by mNGS in these 100 mNGS-positive-only samples, and 118 (118/137, 86.13%) pathogens including 34 (82.93%) G+ bacteria, 49 (89.09%) G− bacteria, and 35 (85.37%) fungi were clinically accepted (**Supplementary Figure 1B**), indicating an outstanding true-positive rate among various sample types and pathogen types.

To further confirm the advantage of mNGS in pathogen detection over culture-based assays, the number of different types of pathogens detected by mNGS and or culture was analyzed. A total of 17 kinds of bacterial species and 10 species of fungi were positive in both mNGS and culture, and 50 bacterial species and 11 fungal species were specifically detected by mNGS only. Generally, mNGS detected much more kinds of bacterial species than culture (**Figure 4B**). In terms of microbial species (**Figure 4C**), *Acinetobacter baumannii*, *Klebsiella pneumoniae*, *Stenotrophomonas maltophilia*, and *Pseudomonas aeruginosa* were the most frequently detected bacteria of both mNGS and culture assays. *Enterococcus faecium*, *Streptococcus pneumoniae*, and *Staphylococcus aureus* were also the bacteria that were detected at a high frequency. For fungi detection, *Pneumocystis jirovecii* was the most frequently detected species of the mNGS assay, which was only detected by mNGS. mNGS and culture tests shared the frequent detection of *Aspergillus* and *Candida*, but mNGS detected more *Aspergillus* than culture, and culture detected a larger number of *Candida* than mNGS (**Figure 4C**).

### 3.5 Pathogen concordance of blood sample and paired respiratory tract samples in the mNGS assay

**Figure 5:**
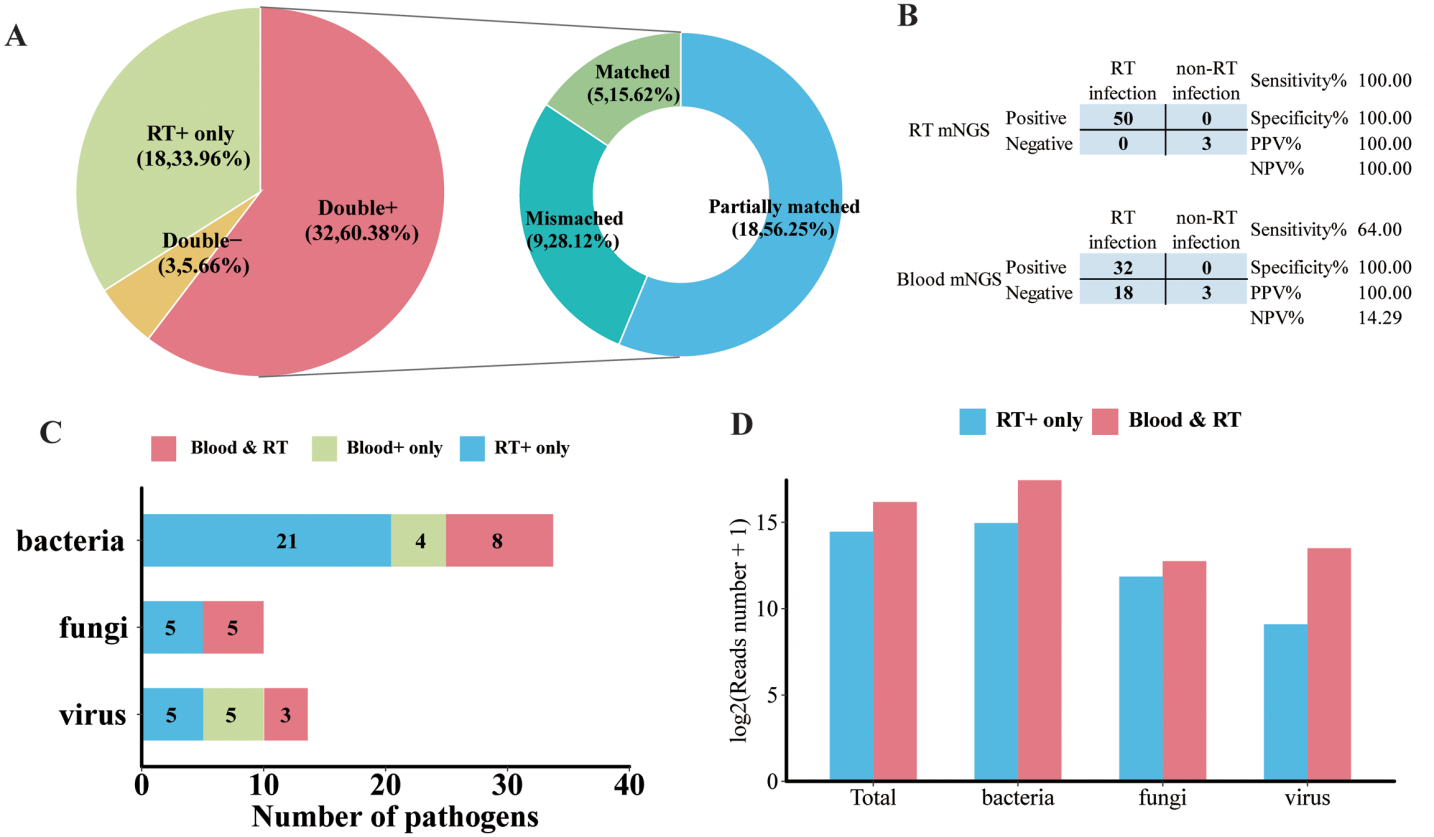
Analysis of pathogen concordance of blood sample and paired respiratory tract (RT) samples in the mNGS assay. **(A)** Pie chart demonstrating the mNGS results in accordance with blood and paired RT as well as the etiology of double-positive samples. **(B)** Contingency tables formatted showing the respective diagnostic performance of RT mNGS and blood mNGS for diagnosing RT infection. **(C)** The number of bacterial pathogens, fungal pathogens, and viral pathogens in the species level that were detected in both blood and paired RT, or only in blood and only in RT. **(D)** The normalized reads number (log2) of total pathogens, bacteria, fungi, and virus in RT that were detected in RT only or RT and blood.

To identify clinical feasibility for blood mNGS as an alternative to the respiratory tract (RT) samples in the mNGS test, a total of 53 pairs of blood and matched RT samples collected from the same patient on the same day were further analyzed. As illustrated in **Figure 5A**, the majority (60.38%, 32/53) of the paired samples were shown to be pathogens positive in both blood and RT by mNGS (double positive). Moreover, mNGS was only positive in RT in 33.96% of samples (RT-positive only). Double negative was found in three pairs, and none were shown to be blood positive but RT negative. Additionally, for double-positive samples, more than half (56.25%, 18/32) shared partial positive pathogens, and five pairs shared identical positive pathogens by blood and RT (**Figure 5A**). Among these 53 patients, 50 were finally diagnosed as RT infection and the remaining 3 patients, whose RT and blood mNGS were both negative, were regarded with non-RT infection. RT mNGS exhibited 100% sensitivity and specificity in diagnosing RT infection; blood mNGS also had a specificity of 100% but the sensitivity was 64% (**Figure 5B**).

In terms of microbial species, more kinds of species were detected in RT than in blood. There were 21 kinds of bacteria, 5 fungi, and 5 viruses detected in the RT sample; 8 species of bacteria, 5 fungi, and 3 viruses were detected in both blood and paired RT samples (**Figure 5C**). Specifically, the majority of *A. baumannii*, *E. faecium, P. aeruginosa, S. maltophilia*, and *S. pneumoniae* along with *Candida albicans* and *Aspergillus flavus* can only be detected in RT samples (**Supplementary Figure 2**). Furthermore, *A. baumannii, K. pneumoniae, P. jirovecii, Human gammaherpesvirus 4* (*EBV*)*, Human betaherpesvirus 5* (*CMV*), and *Human alphaherpesvirus 1* (*HHV-1*) were the most frequently detected pathogens in both blood and paired RT (**Supplementary Figure 2**). In addition, we found that compared with pathogens that can only be detected in RT, pathogens that can be detected in both blood and RT exhibited higher normalized read numbers from RT, regardless of the pathogen type (**Figure 5D**). For example, for pathogens that were detected in both blood and paired RT including *A. baumannii*, *K. pneumoniae, HHV-1*, and *EBV*, they showed relatively higher read numbers in RT than in blood (**Supplementary Figure 3**), suggesting that the microbial nucleic acid in blood may have been circulated from the RT.

## 4 Discussion

mNGS has been extensively applied for discovering causative pathogens and diagnosing infections (Rascovan et al., 2016; Moustafa et al., 2017; Blauwkamp et al., 2019; Chiu and Miller, 2019; Duan et al., 2021). In the present study, we explored the applications and differences between traditional culture methods and mNGS in adult patients with suspected infections via a large-scale sampling. To this end, a total of 511 samples including BALF, blood, sputum, CSF, and other sample types from 361 patients with suspected infection were included. Clinical diagnosis recognized 51.86% of these samples to be pathogen positive, and the clinically accepted rate for mNGS positive result was 81.07%. Among these samples, a total of 428 specimens were subjected to both culture and mNGS testing. We then systematically evaluated the clinical reliability of mNGS in diagnosing infections, as compared to traditional cultures. It was shown that mNGS had superior sensitivity and a clinically accepted rate compared to culture assay with reference to clinical diagnosis. Additionally, the pathogen concordance of blood sample and paired RT samples in mNGS assay from 53 pairs were analyzed, indicating that blood and RT largely shared positive pathogens especially for pathogens with abundant read numbers in RT.

The traditional clinical model for diagnosing infections involves a doctor making a differential diagnosis followed by a series of tests to try to identify the pathogen (Khare et al., 2014; Leber et al., 2016; Cheng et al., 2018). Herein, the clinical diagnosis of each sample was retrospectively made according to not only all routine microbiological tests, but also mNGS assay. Consequently, among all 511 samples, 51.86% were clinically diagnosed as pathogen positive, contributing to 211 of 361 patients (58.45%) who were diagnosed with definite IDs. This positive rate was relatively lower than that of previous studies (Miao et al., 2018; Duan et al., 2021), which may be explained by the fact that about one-third of patients included more than one sample, resulting in a significant number of samples from non-infected sites being sent for testing. Another explanation could be that we enrolled a larger scale of cases with suspected infections rather than just those with confirmed pathogens. Among all sample types, consistent with mNGS results, the highest positive rate for clinical diagnosis was obtained from sputum, followed by BALF; both were from RT. This was expected, considering that RT samples accounted for approximately 40% of all samples and the majority of patients were diagnosed as having respiratory system infection.

In comparison to culture assay, although viruses detected by mNGS were all eliminated, mNGS showed remarkable higher sensitivity (89.08% vs. 56.72%), similar to that reported for mNGS in other studies in adult or pediatric patients (Miao et al., 2018; Tao et al., 2022). After combining the mNGS and culture results, sensitivity increased to 94.94%, also much superior to that of another study (Liu et al., 2023). The outstanding sensitivity in this study may be attributed to the influence of some special samples including tissue, sputum, and other samples, which usually come from infected foci, therefore exhibiting nearly 100% sensitivity by mNGS. Specific to different sample types, some viruses such as *varicella zoster virus*, *Human alphaherpesvirus 2*, and *JC polyomavirus* were clinically recognized as the causative pathogens in CSF but cannot be detected by culture and were excluded in mNGS, leading to the relative low sensitivity in CSF samples (Qu et al., 2022).

When it comes to the true positive results of culture and mNGS, it should be noted that although both clinical diagnosis and culture or mNGS regarded one sample as pathogen positive, whether the pathogens detected by culture or mNGS were consistent with that of clinical diagnosis was inconclusive (Wang et al., 2022a). To clearly address this problem, we introduced the definition of clinically accepted and unaccepted results of mNGS and culture based on the retrospective clinical diagnosis of each sample. For a clinically accepted positive result, at least one detected pathogen should be clinically diagnosed as causative pathogen. We found that the positive result of culture was more likely to be clinically accepted than a negative one, whereas an opposite trend was observed in the mNGS assay. Notably, with both mNGS and culture results, their positive results achieved a favorable clinically accepted rate. In other words, for mNGS- or culture-positive samples, approximately 95% of samples contributed to the identification of causative pathogens. Additionally, we demonstrated that for culture-negative but mNGS-positive samples, approximately 90% of positive results regardless of sample types, as well as detected pathogens, were clinically accepted. Culture-negative infections are challenging to diagnose, and mNGS has been extensively reported to be a powerful tool for identifying pathogens missed by culture (Wang et al., 2020; Tan et al., 2022; Wang et al., 2022b; Zhang et al., 2023). Consistently, our study indicated that mNGS has a superior diagnostic accuracy for detecting suspected infections and may be particularly useful for culture-negative cases.

One of the most common queries about the use of mNGS diagnostic protocols in clinical practice is what kind of sample is best for mNGS testing. Typically, when it was feasible, the site of primary infection yielded the most informative samples for diagnostic sequencing. In traditional bacterial or fungal culture, the most often utilized sample types are blood and RT samples like sputum and BALF. However, because of its extremely poor yield, studies have revealed that blood culture should not be recommended, even in cases of severe pulmonary infections (Afshar et al., 2009; Zhang et al., 2019a). In contrast, it has been demonstrated that mNGS may effectively identify bacteria in blood samples associated with pulmonary infections (Farnaes et al., 2019; Hammarström et al., 2019). Herein, we also investigated the consistency of pathogens detected from blood and paired RT after limiting our inclusion criteria as the sampling interval should be less than 24 h for one patient. Although blood mNGS showed an inferior sensitivity in diagnosing RT infection, we found that for paired blood and RT samples from the same patients, more than half (60.38%) were double positive. Moreover, for blood samples whose mNGS results were both positive in RT samples, 56.25% of samples shared partial pathogens. Further analysis demonstrated the high consistency of pathogens such as *A. baumannii*, *K. pneumoniae, P. jirovecii, EBV*, and *CMV* detected from blood and RT by mNGS. In addition, it was observed that blood mNGS detected more viruses than RT overall, which implied that viruses were more likely to shed into blood compared with bacteria or fungi. On the other hand, the positive blood mNGS result for viruses might also indicate viruses reactivated from other organs or blood (Worth et al., 2016; Yokoyama et al., 2020). Furthermore, we observed that for pathogens that can be detected in blood in addition to RT, their reads were more abundant in RT than pathogens that can only be detected in RT. Meanwhile, the number of microbiological reads detected from the blood samples was relatively lower than that found in RT by mNGS, suggesting that pathogens with more abundant microbiological reads in primary infection sites prefer to shed into blood. It has been demonstrated that the number of unique reads of pathogens was associated with specimen collection time and disease severity (Ai et al., 2018; Zhang et al., 2019b; Li et al., 2020). The high consistency of the major pathogen species, especially those with abundant reads from the RT and blood samples by mNGS testing, suggested, to a large extent, that the pathogens may be transmitted from the lung to the bloodstream in patients who suffered from severe pneumonia. Therefore, the application of mNGS for the simultaneous detection of both RT and blood samples may be promising for identifying microorganisms in patients with suspected infection and guide clinicians regarding antimicrobial treatments; however, the use of blood for RT and the conditions for which it is appropriate are still unclear.

Our study is not without limitations. Firstly, although we enrolled a large number of samples, nearly 100 samples lacked paired culture results owing to the restriction of sample type. Secondly, the clinical benefits, such as the impact on antimicrobial usage, hospital days, and the prognosis of the patients receiving mNGS detection, await further investigation. Thirdly, this study did not collect sufficient clinical characteristics, hampering the establishment of predictive models to resolve the cutoff of clinical indicators and the optimal timing of blood mNGS as an alternative for RT samples. Further studies need to be carried out. Finally, a prospective and observational study could provide more convincing evidence in terms of the diagnostic performance and clinical acceptance of mNGS in IDs in real-life clinical practice.

Collectively, mNGS had a higher sensitivity and clinically accepted rate than culture among various sample types from infectious sites. For patients with suspected infection, the mNGS assay appears to offer complementary clinical value for resolving negative or ambiguous culture results, by helping identify the causative pathogens. Additionally, we demonstrated the favorable consistency of pathogens detected from paired RT and blood samples, indicating that both blood and RT mNGS may aid in the identification of pathogens for respiratory system infection.

## Data Availability

The datasets presented in this study can be found in online repositories. The names of the repository/repositories and accession number(s) can be found below: https://ngdc.cncb.ac.cn, PRJCA027968.
