# Supplementary material for: Diagnostic performance of metagenomic sequencing in patients with suspected infection: a large-scale retrospective study
Source: Front Cell Infect Microbiol. 2024 Sep 6;14:1463081. doi: 10.3389/fcimb.2024.1463081 (PMC11412945; doi:10.3389/fcimb.2024.1463081)
Supplement: Supplementary file 1 [file DataSheet1.docx]

Supplementary Material

**Diagnostic Performance of Metagenomic sequencing in Patients with Suspected Infection: A large-scale Retrospective Study**

**Supplementary table**

Supplementary Table 1. Scenarios for clinically accepted and clinically unaccepted of mNGS/culture result.

| Definition | Description |
| --- | --- |
| Accepted | mNGS/culture positive, clinical diagnosis positive, and at least one detected pathogen was clinically diagnosed as causative pathogen |
|  | mNGS/culture negative, clinical diagnosis negative |
| Unaccepted | mNGS/culture positive, clinical diagnosis negative |
|  | mNGS/culture negative, clinical diagnosis positive |
|  | mNGS/culture positive, clinical diagnosis positive, but none detected pathogen was clinically diagnosed as causative pathogen |

**Supplementary figures**

**
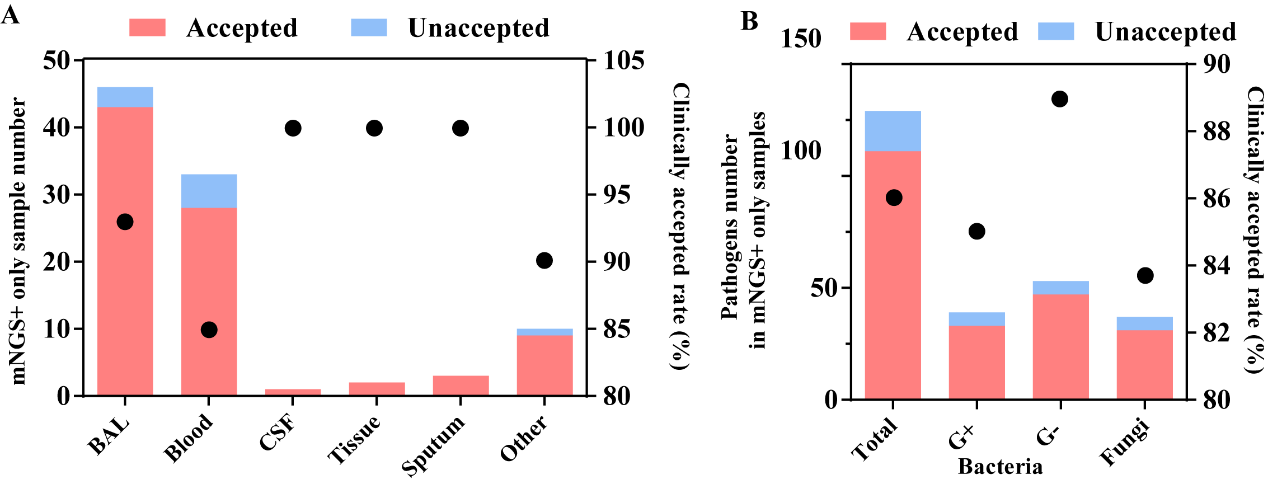
**

Supplementary Figure 1. The distribution of clinical acceptance of mNGS-positive culture-negative samples. A, sample type distribution and corresponding clinically accepted rate of mNGS positive only samples (n=95). B, positive pathogen types and corresponding clinically accepted rate in mNGS positive only samples.


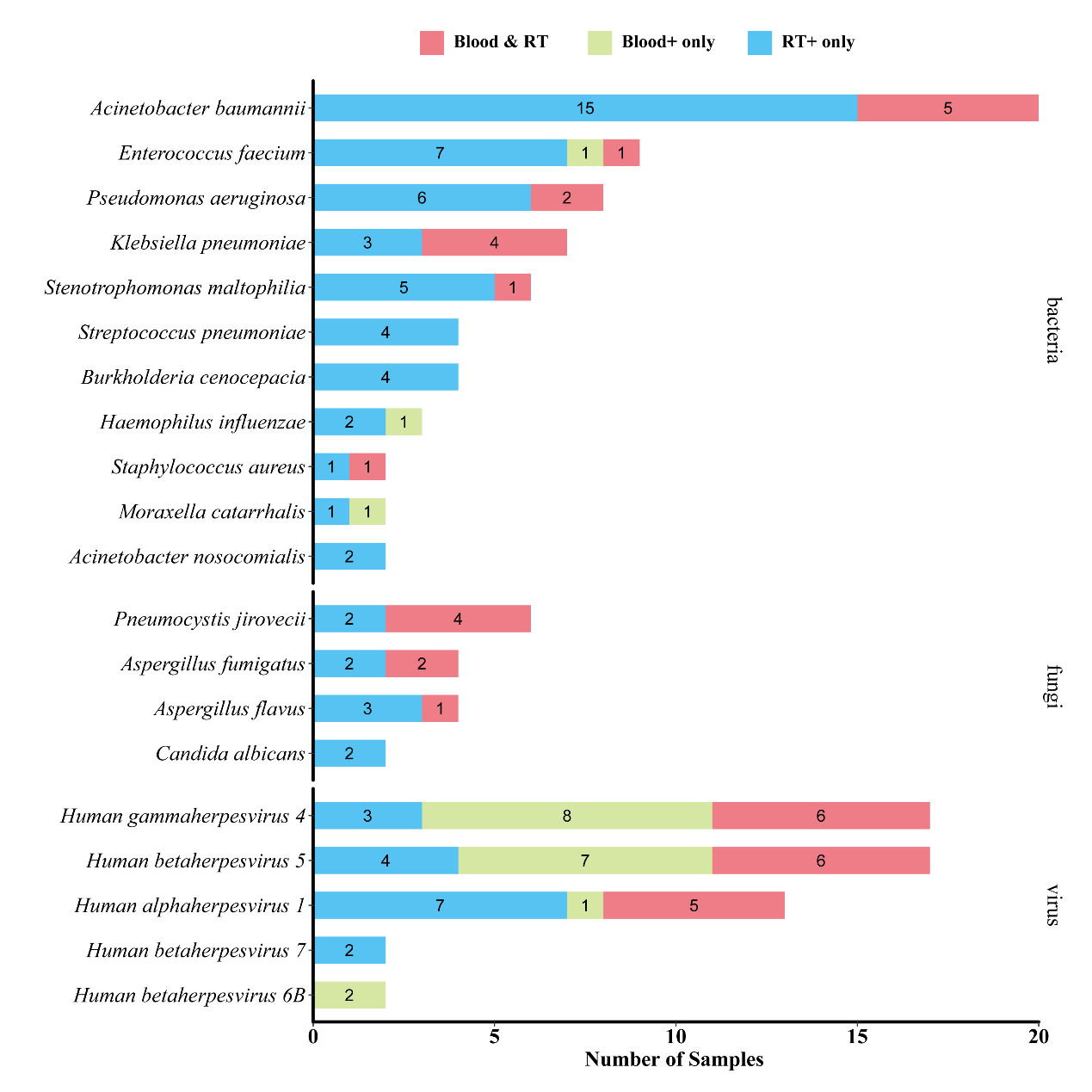


Supplementary Figure 2. The pathogens profiles that were detected in both blood and paired RT, or only in blood and only in RT by mNGS. The number of bacterial pathogens, fungal pathogens, and viral pathogens in the species level.


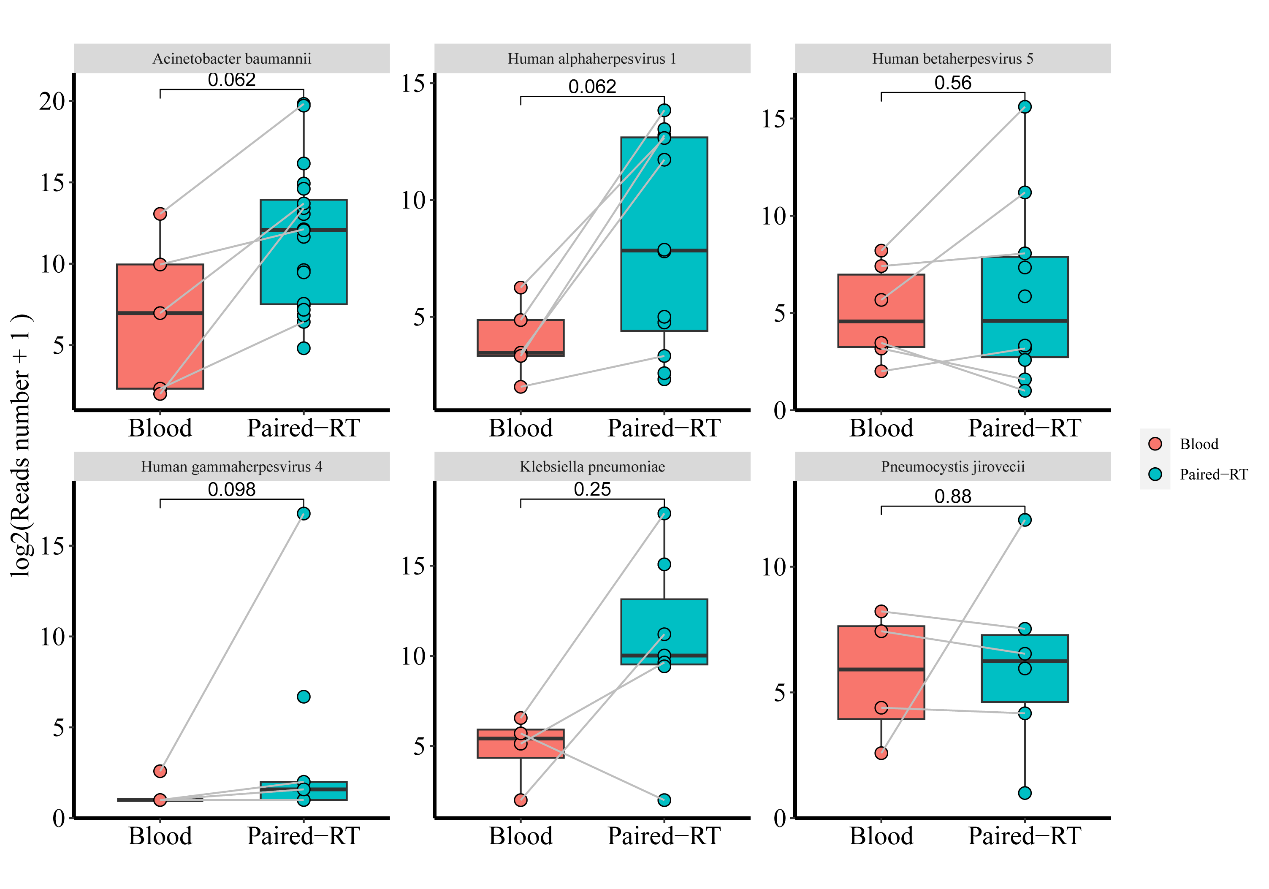


Supplementary Figure 3. Comparisons for reads number of illustrated microbes including *Acinetobacter baumannii*, *Human alphaherpesvirus 1, Human betaherpesvirus 5, Human gammaherpesvirus 4, Klebsiella pneumoniae* and *Pneumocystis jirovecii* in blood and paired RT.
